# Role of Magnesium Perrhenate in an Oil/Solid Mixed System for Tribological Application at Various Temperatures

**DOI:** 10.3390/ma11091754

**Published:** 2018-09-18

**Authors:** Junhai Wang, Ting Li, Tingting Yan, Lixiu Zhang, Ke Zhang, Xin Qu

**Affiliations:** School of mechanical Engineering, Shenyang Jianzhu University, No. 9 Hunnan East Road, Hunnan District, Shenyang 110168, China; liting@sjzu.edu.cn (T.L.); yantt1120@163.com (T.Y.); zhanglixiu@sjzu.edu.cn (L.Z.); zhangke@sjzu.edu.cn (K.Z.); quxin16333@163.com (X.Q.)

**Keywords:** magnesium perrhenate, oil additive, various temperatures, tribology

## Abstract

Magnesium perrhenate used as a lubricating additive was prepared by an aqueous solution method in this paper, and was suspended in a base oil poly alpha olefin (PAO6) with the aid of surface active agents (SA). The thermal stability of the mixed oil with/without magnesium perrhenate and surface active agents was investigated by thermogravimetry testing. The influences of magnesium perrhenate as solid lubricating additive on the extreme pressure performance and the friction-reducing properties over a wide temperature range of the mixed lubricants were determined by four-ball tests and ball-on-disc frictional tests for the commercially available silicon nitride ball and a Ni-base superalloy frictional pair. The results revealed that the added magnesium perrhenate did not obviously affect the thermostability and oxidation resistance of the base oil. Meanwhile, it minimized the coefficients of friction and wear scar diameter to a certain extent in the four-ball experimental conditions. Ball-on-disc rubbing tests suggested the mixed oil had a similar lubricating performance to the base oil below the decomposition temperature point. The most significant advancement was the impressive antifriction improvement at the high temperature range, while the friction coefficients of the oil containing magnesium perrhenate compound were obviously below that of the base stock. This better tribological performance of the mixed lubricant was attributed to the native shear susceptible property and chemical stability of magnesium perrhenate under high temperature conditions, which could form an effective durable and stable antifriction layer with the oxides from the superalloy matrix, thereby decreasing the friction in the high-temperature environment.

## 1. Introduction

Completing and maintaining low-friction during a board range of temperatures is a challenging task for the tribology community. Fast developments in aviation, nuclear industry, materials processing, advanced machines and other areas of high technology have led to higher requirements for lubricants with low coefficients of friction with a broad temperature scope, because frictional parts are often exposed to cyclic environments with alternating conditions of room and high temperature application [1,2,3,4,5]. Lubricants with fine tribological properties at various temperatures are largely required, because increased friction and wear would lead to inevitable losses of energy and materials, and subsequently reduce the efficiency of the mechanical system. In recent decades, to fulfill antifriction over a broader range of temperatures, the various types lubricating materials have appeared and been applied under special operating conditions. However, lubricating materials are highly sensitive to temperature, and perform the lubrication function within a narrow temperature interval [6,7]. Fluid lubrication is always applied below 300 °C, and will lose proper lubrication quality above that temperature by reason of oxidative decomposition [8]. As candidates of solid lubricating materials, some layered structures of lubricating agents, such as graphite and MoS_2_, can offer low-friction owing to their shear structure with a preferred easy shear parallel to the basal planes of the crystallites. Nevertheless, the maximum serve temperature is limited to 500 °C in air because of their inadequate oxidation resistance [9]. Although the fluoride, oxides, and rare earth components as efficient solid lubricants can serve even higher temperature, they are usually brittle and work ineffectively on lubricating at low temperatures [10]. At present, despite intensive exploration and developmental achievements in the past decades, it is difficult or impossible for single kind of lubricant to realize the continuous friction-reduction requirement over a broad temperature range. At present, the self-lubricating bulk composite materials and self-adaptive lubricating composite layer have been applied as a solution for the lubrication problem over a wide range of temperatures via multi ingredient design, and utilize the lubricating feature of the different ingredients at various temperature stages to minimize friction. However, the mechanical properties of bulk materials are deteriorated when excessive lubricating phases existed. The lubricating layers are expensive to prepare and are difficult to maintain. Furthermore, the solid materials always possess a relatively high friction coefficient at the low temperature range. All these defects have limited the development of self-lubricating bulk materials and lubricating layers over a wide temperature range. Therefore, it is an effective and practical measure to maintain lubrication covering a broader temperature range to use the multi-components hybrid mode with fluid lubrication for the low-temperature stage and solid lubrication for the high-temperature zone. The design and preparation of solid lubricant is vital to successful antifriction in the hybrid mode, since it should produce friction-reduction at the moment of oil oxidative decomposition, and keep lowering the friction over a range of high temperatures. In addition, it is key to ensure that the solid lubricant does not deteriorate the friction-reducing and antiwear nature of the base stock at low temperatures.

As mentioned above, many trials have been made to discover ways of feasible alteration of solid lubricating materials. Double metal oxides as inorganic salts are potential candidates for consideration in virtue of their low Mohs hardness compared with that of single oxides and thermal softening that make them shear susceptible at the high temperature range. Additionally, they are also likely to be promising candidates to solve the challenge of high-temperature friction reduction owing to their chemical stability in air. Many literature reports show that some chromates, molybdates, tungstates, and vanadates have attracted much attention and been applied as high temperature solid lubricants via direct preparation methods or tribochemical reaction over the course of friction at elevated temperatures [11,12,13,14,15,16,17,18,19,20,21]. All these double metal oxides provide fantastic lubrication performances at elevated temperatures, and give a guide as to the usage of them to explore boundary lubrication. In addition, as alkaline earth metal element, magnesium or magnesium compounds have been widely used as lubricating additives to aid friction reduction during the friction process. It is well known that talcum powder has the lowest Mohs hardness in nature, and this contains plenty of the magnesium element. The research outcomes on the lubricating results of composites containing magnesium element are impressive. Qi et al. synthesized magnesium hexasilicate and a serpentine compound used as oil additive, and the tribological properties at elevated temperatures were investigated. Both of the magnesium compounds can generate protective layers with metal matrix, thereby reducing the friction [22,23]. Wang et al. prepared the Mg-Al-CO_3_ LDH (layered double hydroxide) compound and found that it possessed excellent properties of friction and wear reduction as lubricating oil additive [24].

In this paper, magnesium perrhenate, as a novel inorganic double metal oxide, was synthesized, characterized, and its tribological performances as a potential lubricating additive in synthetic oil for Si_3_N_4_/GH4169 investigated at various temperatures from 25 °C to 600 °C in air atmosphere. The purpose of this work was to achieve a comprehensive insight on the behavior of magnesium perrhenate as lubricating additive, with special emphasis on the role of ambient temperature on the characteristics of the friction-reducing layer and the relevant friction behavior of the coupling. Meanwhile, the synergy effect and mechanism of PAO containing Mg(ReO_4_)_2_ additive on the tribological attributes of the rubbing surface from 25 °C to 600 °C in air atmosphere were analyzed and discussed in detail.

## 2. Materials and Methods

### 2.1. Materials Preparation

The raw materials used in synthesizing the new product were rhenium powders (>99.999% in purity, Zhonglai Science and Technology Co. Ltd., Changsha, China), hydrogen peroxide (H_2_O_2_, 30% concentration, Sinopharm Chemical Reagent Co. Ltd., Shanghai, China) and magnesium hydroxide powders (>99.5% in purity, Sinopharm Chemical Reagent Co. Ltd., Shanghai, China). At the beginning, the 0.003 mol rhenium powders were weighed, then added rapidly into 20 mL hydrogen peroxide solution. The hybrid compounds were continuously stirred and lots of bubbles were emitted, while the solution color changed from grey to transparent. The perrhenic acid (HReO_4_) was continued until free of bubbles. In the subsequent step, 0.131 g of magnesium hydroxide powders were added to freshly prepared HReO_4_ solution with constant mixing. At the end of the reaction, the lower sediment was discarded using qualitative filter paper. Then, the obtained reactant was maintained in the water bath at a temperature of 65 °C until a white layer of crystalline material was generated. The resulting product (Mg(ReO_4_)_2_) was removed from the water bath and dried in a vacuum oven for 2 h at a temperature of 200 °C.

The PAO6 (Hongcheng Chemical Co. Ltd., Shenyang, China) was chosen as base oil due to its high oxidative resistance temperature, excellent viscosity-temperature characteristics, high flash point, and low-volatility. The typical physical parameters of PAO6 aree listed in Table 1. Fine dispersion stability was the crucial factor for the practical application of oil additive. The synthesized Mg(ReO_4_)_2_ was a kind of inorganic particle, and the particles aggregated after drying. Thus, it was difficult to directly disperse them into base oil. Instead, an indirect dispersion method was employed. The polyoxyethylene octyphenyl ether (OP10, C_8_H_17_C_6_H_4_O(CH_2_CH_2_O)_10_H, Sinopharm Chemical Reagent Co. Ltd., Shanghai, China) and cetyltrimethyl ammonium bromide (CTAB, C_16_H_33_(CH_3_)_3_NBr, Sinopharm Chemical Reagent Co. Ltd., Shanghai, China) were selected as surface active agents (SA). The preparation process of 5 g of PAO with 0.5 wt% Mg(ReO_4_)_2_ additive was used as illustration. The 0.025 g of Mg(ReO_4_)_2_ compounds were fully dissolved in water forming a supersaturated solution first. Then, a weight of 0.05 g of surfactants (SA, 0.045 g of OP10: 0.005 g of CTAB) were added into the 5 g PAO oil using ultrasonic oscillation method for 30 min to reduce surface tension of the base oil and establish a hydrophilic–lipophilic balance state. Afterwards, the supersaturated aqueous solution was added into the base oil containing SA and continuously stirred at a rotation velocity of 1500 rpm for 10 min at a temperature of 140 °C in an oil bath till no obvious bubble (boiling water) was released. The base oils blended with various contents of Mg(ReO_4_)_2_ additive using the same dispersion technology were applied to complete a series of frictional tests.

The phase composition was investigated via an X-ray diffraction (XRD, Shimadzu Corporation, Tokyo, Japan) tester with Cu Kα radiation (λ = 0.1546 nm) at a scanning speed of 5°/min in the 2θ range of 10–90°. The micro-morphology of the synthesized product was observed using a FEI INSPECT 50 scanning electron microscopy (SEM, FEI Corporation, Hillsboro, OR, USA) equipped with an energy dispersive spectrometer (EDS). Differential thermal analysis/thermogravimetry (DTA/TG,) analysis was determined in the ambient atmosphere with a SETSYS Evolution18 thermal analyzer (SETARAM Corporation, Caluire, France). The rate of heating addition was 10 °C/min under a flow of air with speed of 30 mL/min. To evaluate the suspended stability of magnesium perrhenate in the base oil, a UV-3900 visible–ultraviolet spectrophotometer (Shimadzu Corporation, Tokyo, Japan) was used to investigate the transmittances of PAO containing Mg(ReO_4_)_2_ at different stages (0 h, 3 h, 6 h, 12 h, 24 h, 36 h, 48 h and 60 h). The RO-water (filtered water through a reverse osmosis membrane) was used as reference solution, and its transmittance was defined as 100%.

### 2.2. Four-Ball Test

The four-ball wear tests were evaluated on the WWM-1 four-ball tester (Shunmao Instrument Co. Ltd., Jinan, China) at 392 N (the mean hertz contact pressure was around 1230 MPa) and 1450 rpm at room temperature for 30 min according to the ASTM D4172 standard [25]. The test balls with a diameter of 12.7 mm in this experiment were made up of Cr alloy steel, having a hardness between 59–61 HRC. Corresponding tests were carried out for every concentration and three repeat surveys were executed, and the mean friction coefficient values and wear scar diameters (WSD) of the lower ball are reported in this paper. The original and final temperatures of the oil mixtures were detected by a thermometer attached to the four-ball tester. The friction coefficients were automatically recorded by computer using a data-acquiring system linked to the tester, and a VHX-1000E super depth field optical microscope (Keyence Corporation, Osaka, Japan) was employed to measure the WSDs. The surface morphologies of wear scars were observed by an FEI INSPECT-F50 SEM instrument.

### 2.3. Tribological Tests over a Wide Temperature Range

**Figure 1:**
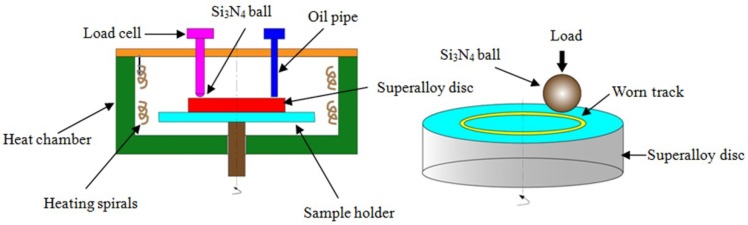
The schematic drawing of the MFT 5000 ball-on-disc experimental device.

The tribological measurements over a broad range of temperatures were evaluated on the Rtec MFT 5000 multifunction tribotester (Rtec Instrument Corporation, San Jose, CA, USA) with a ball-on-disc configuration, and the schematic diagram of test device is presented in Figure 1. An Si_3_N_4_ ball with a diameter of 6.35 mm (roughness < 0.02 μm) and a GH4169 Ni-based alloy disc (chemical composition: ≤0.08 wt% Carbon, ≤1.0 wt% Cobalt, 2.8–3.3 wt% Molybdenum, 0.3–0.7 wt% Aluminum, 0.75–1.15 wt% Titanium, 17–21 wt% Chromium, 50–55 wt% Nickel and balance iron) with a size of Ø50 mm × 4 mm were used as the friction pair materials for the tribological testing. The GH4169 superalloy disc was ground continuously by different types of abrasive paper and polished with grinding agent to obtain a surface roughness of 0.1–0.2 μm. Both the ball and the disc were ultrasonically cleaned for 30 min by acetone to remove contaminants before the friction test. The tests were carried out at 25 °C, 200 °C, 350 °C, 450 °C, and 600 °C, and each test was held for 8 min. A load of 5 N (the mean hertz contact pressure was around 500 MPa) and a liner velocity of 0.08 m/s were applied for testing parameters. Each of the work conditions was tested three times to guarantee the repeatability and reproducibility of the data, and the mean values of the friction coefficients were reported.

The microstructure and morphologies of the wear track were characterized using an FEI INSPECT-F50 SEM instrument. The ESCALAB250 X-ray photoelectron spectrometer (Thermo Fish Scientific Corporation, Waltham, MA, USA) using the Al Kα line as excitation source at a pass energy of 50 eV, and binding energy of C1s (284.6 eV) was used as reference. The worn track widths of the lower discs were measured by using a VHX-1000E super depth field optical microscope.

## 3. Results and Discussion

### 3.1. Characterization of Mg(ReO_4_)_2_

**Figure 2:**
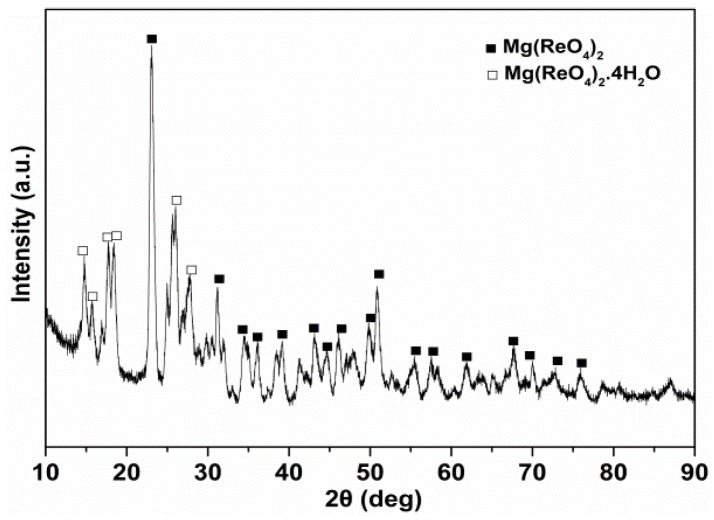
The X-ray diffraction (XRD) pattern of synthesized powder.

**Figure 3:**
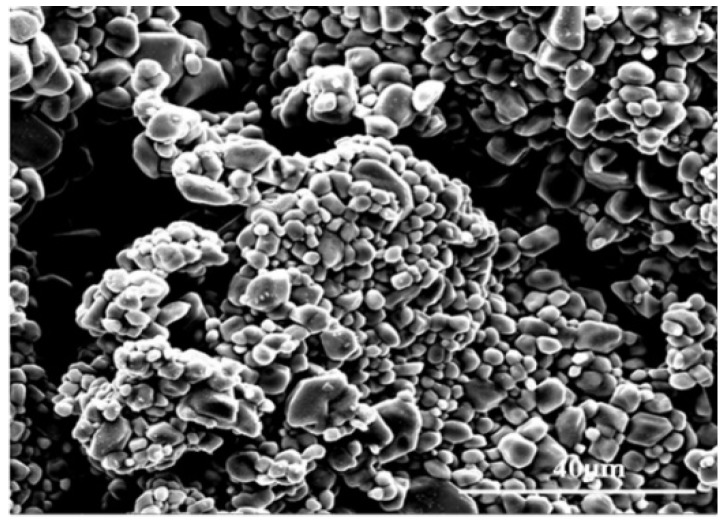
The scanning electron microscope (SEM) image of synthesized powder.

Figure 2 showed the XRD pattern of the synthesized product. The synthesized component was mainly composed of the Mg(ReO_4_)_2_ phase (PDF No. 33-0880) along with a small amount of Mg(ReO_4_)_2_·4H_2_O phase (PDF No. 37-0028) [26]. No other characteristic peaks corresponding to impurity were detected, implying the high purity of the as-synthesized sample. The morphology of the synthesized powders is shown in Figure 3. It was observed that the Mg(ReO_4_)_2_ powders were rounded in shape with a size distribution of 1–6 μm.

### 3.2. Dispersion Stability and Thermal Stability

**Figure 4:**
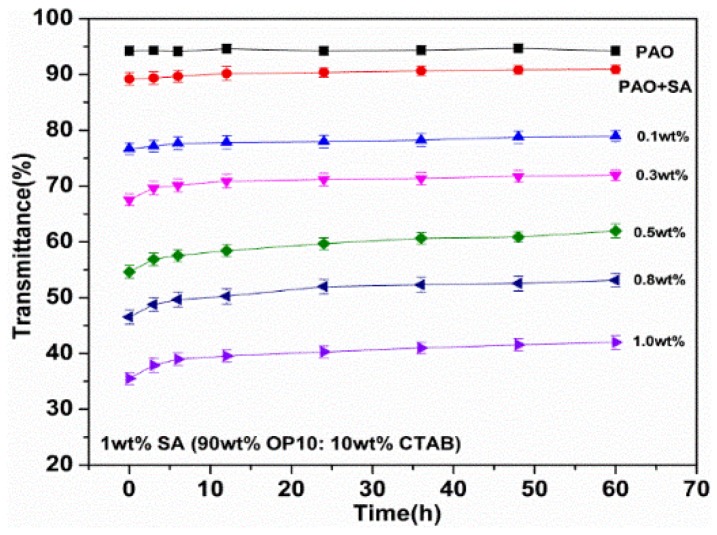
The transmittances of poly alpha olefin (PAO) containing various concentration of Mg(ReO_4_)_2_ additive with the assistance of surface active agents (SA).

To investigate the dispersion of Mg(ReO_4_)_2_ additive in PAO oil, the transmittance of base oil with diverse additive concentrations along with the time change are displayed in Figure 4. The transmittance of all the test specimens declined with increasing contents of Mg(ReO_4_)_2_ additive, indicating that a certain mass of Mg(ReO_4_)_2_ additive was suspended in the PAO oil with the aid of SA. Additionally, the transmittance attained slight variation and achieved a stable tendency over time, indicating that Mg(ReO_4_)_2_ could be well suspended in the hydrophilic–lipophilic balance environment modified by addition of SA.

**Figure 5:**
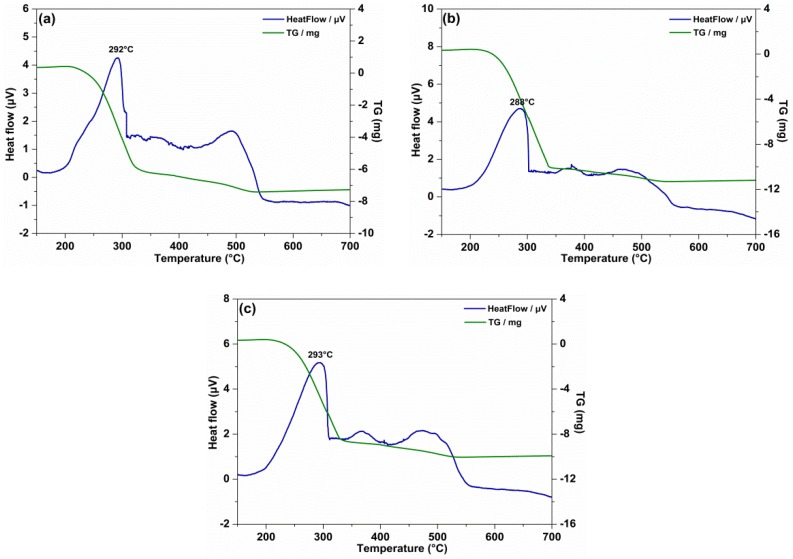
The thermal analysis/thermogravimetry (DTA/TG) curves of different lubricating specimens: (**a**) PAO; (**b**) PAO–SA; (**c**) PAO–SA–0.5 wt% Mg(ReO_4_)_2_.

To evaluate the influence of surface active agents and Mg(ReO_4_)_2_ compound on the thermal stability of PAO base stock, DTA/TG was employed to determine the tolerance of the lubricating materials to thermal-oxidative degradation. Figure 5 shows a distinct weight loss of all the test specimens which appeared in the temperature range 200–320 °C. The distinct exothermic peak of PAO was apparent at 292 °C (Figure 5a), indicating that for the pure oil rapid oxidation took place at that temperature t, and further explained why PAO had a fine thermal stability as a lubricating oil. The exothermic peak location of SA addition was similar to that of PAO (Figure 5b), suggesting that the surfactant itself had a slight effect on the thermal property of base oil. The maximum exothermic peak of PAO containing 0.5 wt% Mg(ReO_4_)_2_ was apparent at around 293 °C because of vigorous oxidation of the organic functional groups of the base oil (Figure 5c). Based on the test results, the addition of a certain amount of organic surfactant and synthesized inorganic additive had no effect on the oxidative stability of base oil.

### 3.3. Four-Ball Test Analysis

**Figure 6:**
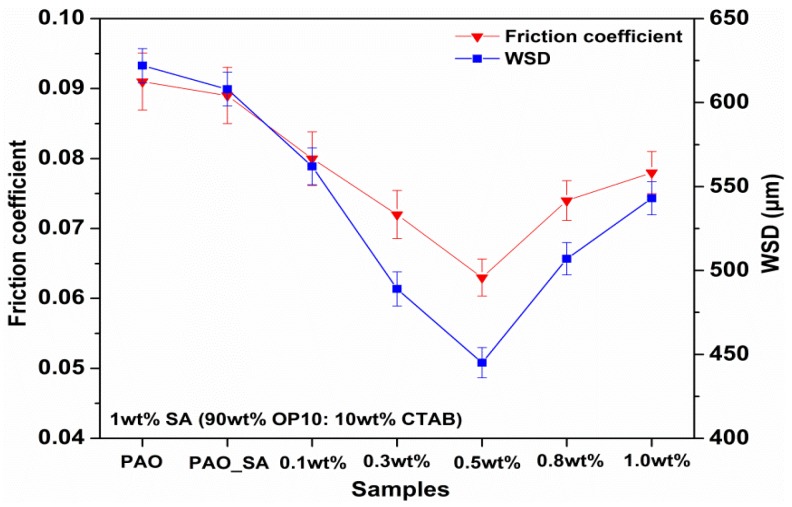
The effect of various concentrations of Mg(ReO_4_)_2_ additive on the average values of friction coefficient and wear scar diameter (WSD).

Figure 6 displays the mean friction coefficient values and wear scar diameters of base oil with various concentrations of magnesium perrhenate under the four-ball test situations. The average friction coefficients of the oil with Mg(ReO_4_)_2_ additive were decreased over the content range of 0.1–0.5 wt%, and attained the minimum value at a concentration of 0.5 wt%. The average value of the friction coefficient was 0.063 at an optimal content of 0.5 wt%, which was reduced by 30.8% in contrast to the value of base oil (0.091). The downward trend of average friction coefficients could have resulted from the generation of deposited tribofilm made up of long-chain molecules from base oil and added Mg(ReO_4_)_2_ additive on the contact surfaces during the test period. Afterwards, the average friction coefficients of the solid/oil mixture increased along with the increasing Mg(ReO_4_)_2_ additive content. Even so, the average friction coefficients of oil with various contents of Mg(ReO_4_)_2_ additive were distinctly lower than that of pure oil in the given test conditions. The variation of WSD values of the lower balls lubricated by diverse additive contents in base stock is also shown in Figure 6. It is clearly seen that the introduction of Mg(ReO_4_)_2_ additive in PAO resulted in a remarkable decrease of WSD values, and the smallest WSD of 445 μm was attained at a content of 0.5 wt%, which was reduced by 28.5% in contrast to that in the lubrication state of PAO only (622 μm). Then, the WSD values increased with the further rise of additive concentration above 0.5 wt%. The reason for the improving friction-reducing capacity of PAO rested on the fact that the Mg(ReO_4_)_2_ additive could be absorbed on the interface to produce a protective film under high contact load, thereby avoiding direct contact of the friction pairs and minimizing friction. Above the optimal content, the WSD values were slightly increased associated with additive concentration increase, which was ascribed to the fact that the agglomeration of the excess Mg(ReO_4_)_2_ additive on the local worn area hampered their uninterrupted supply to the contact interface [27]. This caused the increase in friction, even regional destruction of the protective layer, and thereby led to relatively high wear aggravation with excessive additive concentration.

**Figure 7:**
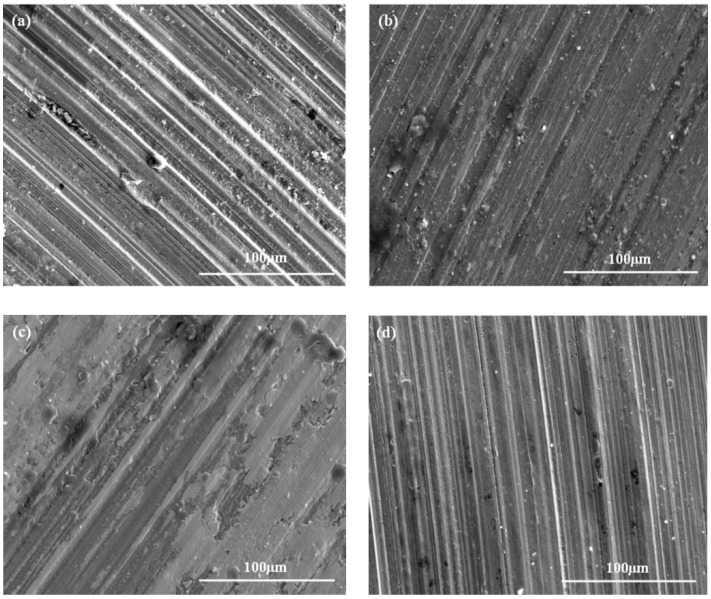
The morphologies of wear scars lubricated by PAO with different contents of Mg(ReO_4_)_2_ additive (**a**) PAO; (**b**) 0.3 wt% Mg(ReO_4_)_2_; (**c**) 0.5 wt% Mg(ReO_4_)_2_; (**d**) 1.0 wt% Mg(ReO_4_)_2_.

For determining the lubricating performance and wear mechanism, the wear scar of the lower ball after four-ball tests in the oil mixtures were observed. Figure 7 displays the morphologies of wear scar of the lower ball under lubrication conditions of PAO oil containing different contents of Mg(ReO_4_)_2_ additive. It is clearly seen that the wear scar under base oil lubrication was seriously damaged with a deeper and wider plough, and some fine debris was distributed in the furrows (Figure 7a), illustrating that severe wear and plow occurred in the test process, which was attributed to the poor lubricating film bearing strength generated by the PAO base stock only. In contrast to that lubricated by pure oil, the wear scars under PAO with Mg(ReO_4_)_2_ additive lubrication were smoother, and the furrows were relatively narrow and shallow (Figure 7b–d). This revealed the synthesized Mg(ReO_4_)_2_ as lubricating additive was effective in reducing wear of the steel–steel friction pair to a certain degree when it was added into the PAO synthetic oil. This might be ascribed to the generation of a positive protection layer on the contact interface.

It was clear that almost all the energy expended in the process of friction emerged in the form of the heat across the contact interface. The friction heat induced temperature rise in the rubbing pairs and the oil mixtures. Thus, the smaller energy expended resulted in less temperature increase. As a result, Table 2 exhibits the effect of the concentration on the temperature rise of lubricating samples. Note that the base oil with 0.5 wt% magnesium perrhenate additive produced the minimum temperature rise compared to the remaining specimens. This was similar to the results of the friction coefficients and WSD results. Furthermore, the temperature rise results demonstrated the excellent tribological property of the Mg(ReO_4_)_2_ component as a lubricating additive function.

### 3.4. Tribological Performances at Various Temperatures

**Figure 8:**
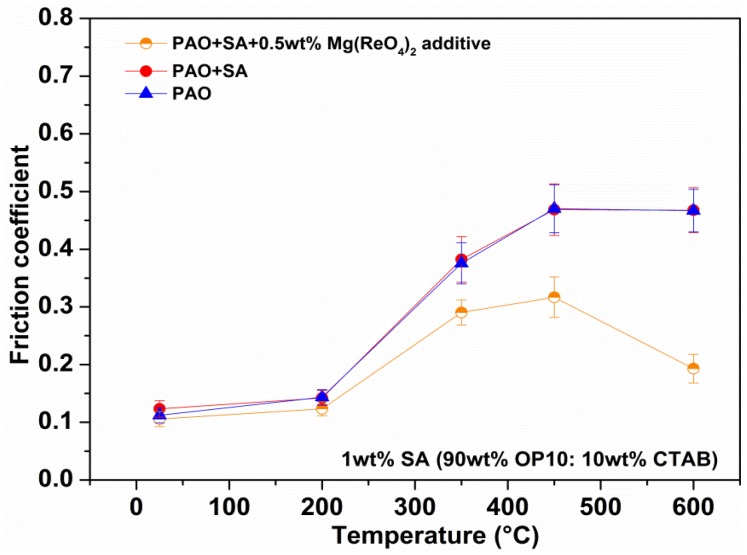
The average friction coefficient values of rubbing pairs lubricated by various lubricants at different temperatures.

Figure 8 presents the average friction coefficients of the Ni-based alloy sliding against a Si_3_N_4_ ball with various lubricating samples at various temperatures. The base oil exhibited a favorable friction-reducing nature with coefficients of friction being in the 0.11 to 0.15 range below 200 °C. However, a distinct increase in friction coefficients of PAO above 350 °C was due to oxidative decomposition, which made the PAO base oil lose its lubricating service purpose. In addition, the carbon residue generated from the high temperature environments would result in increasing wear. Thus, the friction coefficients of pure PAO attained relatively high values of 0.47 at 450 °C and 0.46 at 600 °C, respectively. Additionally, the average values of the friction coefficient of base oil with added surfactants were similar to that of pure oil under test conditions, which illustrated that the added surfactants did not influence the antifriction function of PAO oil for the whole service processes. In comparison with base oil, the added Mg(ReO_4_)_2_ additive could slightly enhance the lubricating property of base oil below 200 °C. The friction-reducing effect under ball-on-disc configuration was not as obvious as that under the four-ball test, which is related to the different friction test parameters and experimental conditions. Nevertheless, the friction coefficient of base oil containing Mg(ReO_4_)_2_ additive remarkably attained a minor value of about 0.29 when the temperature reached 350 °C. With the temperature further increasing to 450 °C and 600 °C, the average friction coefficients of PAO with Mg(ReO_4_)_2_ additive were significantly lower than that of pure oil, and the value decreased to the minimum point about 0.19 at 600 °C within a high temperature range. The main reason for this is explained by the fact that the Mg(ReO_4_)_2_ additive being a novel elevated temperature solid lubricant, it might have a positive role in minimizing the friction.

**Figure 9:**
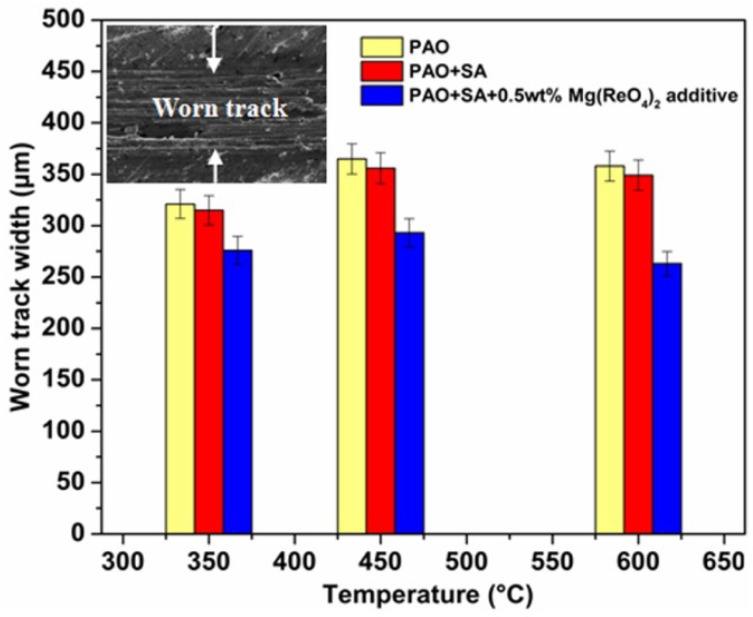
The worn track values of superalloy lubricated by different lubricants at elevated temperatures.

Figure 9 shows the worn track widths of lower GH4169 superalloy discs sliding against Si_3_N_4_ ball lubricated by diverse samples at elevated temperatures. The worn track width values of superalloy with pure base oil lubrication were the largest among all the test lubricants in the range of 350–600 °C, suggesting that the lubricating function of base oil deteriorated in the elevated temperature environment. Meanwhile, the oil with added SA possessed the similar values of worn track width and obtained the same trend at identical temperature conditions. In contrast, the mean width values of wear track under oil with 0.5 wt% Mg(ReO_4_)_2_ mixture lubrication were 276 μm, 293 μm and 263 μm at 350 °C, 450 °C, and 600 °C, respectively, which were lower than that of worn track lubricated with base stock alone or oil containing surfactants to varying degrees. The reason for this might be that a protective layer containing Mg(ReO_4_)_2_ additive was formed on the friction surface by deposition and adhesion to the contact interface under the mixing effect of stress and frictional heating, which was sheared easily and could be restrained in direct contact between rubbing pairs, and thus mitigate the wear of the metal surface. In general, the increasing worn track width resulted from the lager plastic deformation of the worn surface [28]. Furthermore, the deformation due to friction and the succeeding microstructure variation below the contact interface were responsible for the friction coefficients increasing. Overall, comparing the wear experienced on the worn surfaces, the Mg(ReO_4_)_2_ additive decreased plastic deformation during the friction tests, indicating it possessed a superb friction-reduction function at the high temperature environment.

**Figure 10:**
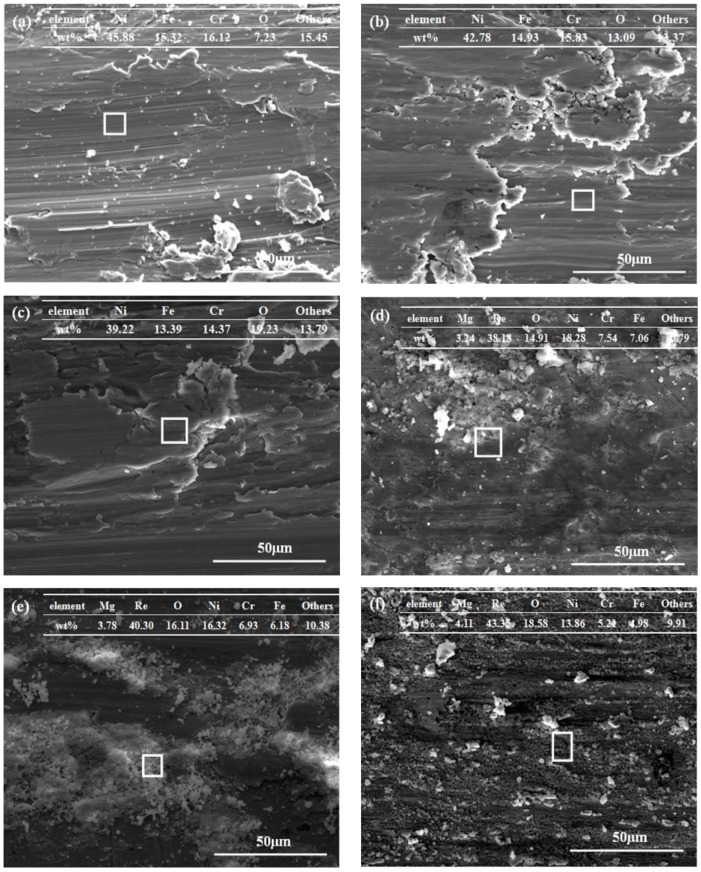
The morphologies of worn surfaces lubricated by different lubricating mediums at various temperatures (**a**) 350 °C, PAO; (**b**) 450 °C, PAO; (**c**) 600 °C, PAO; (**d**) 350 °C, PAO_0.5 wt% Mg(ReO_4_)_2_; (**e**) 450 °C, PAO_0.5 wt% Mg(ReO_4_)_2_; (**f**) 600 °C, PAO_0.5 wt% Mg(ReO_4_)_2_ and the energy dispersive spectrometer (EDS) results of marked areas.

To analyze the tribological performance and wear mechanisms of oil with or without Mg(ReO_4_)_2_ additive, a scanning electron microscope was employed. Figure 10 shows the worn surfaces of superalloy disc under diverse samples lubrication at various temperatures. It can be seen from Figure 10a, that the micro-grooves and detachments of the deformed surface appear at 350 °C, indicating that the base oil has lost lubricating function. Additionally, small pits and some debris were also observed on the worn surface, which illustrates that the main wear mechanism was micro-plough. At 450 °C, severe deformation and delaminations evidently emerged on the rubbing surface, which was responsible for the increased coefficient of friction at that temperature (Figure 10b). As the temperature reached 600 °C, the worn track displayed severe damage with evident appearance of plastic deformation (Figure 10c). In addition, delamination and plowed grooves existed on the worn surface. This indicated that the major wear mechanism of the rubbing pair was characterized by adhesive wear. The EDS results after friction tests with pure PAO at different temperatures showed that nickel, iron, chromium, oxygen elements existed on the worn surface, as well as less amounts of elements such as carbon. Especially, the contents of oxygen on the worn surface increased with the rise of temperature, which was attributed to diffusion promotion of the oxygen in pace with the elevated temperature. This indicated that the oxygen easily reacted with the native elements of the superalloy, and diffused towards the worn surface to form an oxide layer. Nevertheless, this oxide layer was hard and brittle and was easily removed by the shearing force in the process of the friction test [29]. Consequently, it was hard to develop a stable protective layer with the function of oxidative inhibition, avoiding direct contact of the rubbing pair, and making no contribution to friction-reduction. In contrast, the worn surface under PAO containing Mg(ReO_4_)_2_ additive lubrication was much smoother than that lubricated by pure oil at 350 °C, and a continuous layer was covered on the metal surface with fine wear debris, which was ascribed to generation of protective layer that could effectively minimize the wear of the counterface (Figure 10d). To investigate the composition distribution on the worn surface, EDS analysis was conducted. The result suggested that the major elements appearing were Mg, Re, O, Ni, Fe, Cr, et al., which were ingredients of the Mg(ReO_4_)_2_ additive and the superalloy. This indicated that the addition of Mg(ReO_4_)_2_ additive participated in the generation of the layer, and was responsible for minimizing friction. At 450 °C, the morphology of the worn surface under PAO with Mg(ReO_4_)_2_ additive lubrication was characterized by a continuous dense layer, covering most regions with shallow delamination and plastic plow on the local area (Figure 10e). The EDS result showed that a large amount of Mg(ReO_4_)_2_ and partially oxidized metallic Ni, Fe and Cr mixture smeared and uniformly distributed over most of the worn surface could weaken attraction between the friction pair with increasing temperature. At 600 °C, a compact layer with some wear debris developed on the worn track with the effect of frictional force and load (Figure 10f). Although the EDS result was always only used as the basis of qualitative analysis, it was confirmed that the major elemental compositions were rich in Mg, Re, and O, suggesting that the composition of Mg(ReO_4_)_2_ additive was maintained in the layer, which functioned as wear-resistant holder to offer a bearing capacity. In addition, this layer was made up of complex solid lubricants containing an easy slip structure that produced lower coefficient of friction.

**Figure 11:**
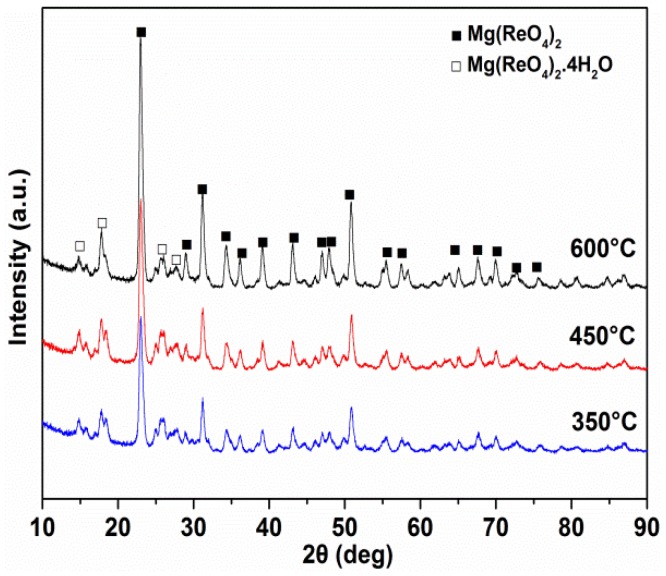
The XRD patterns of Mg(ReO_4_)_2_ with heat treatment at different temperatures.

**Figure 12:**
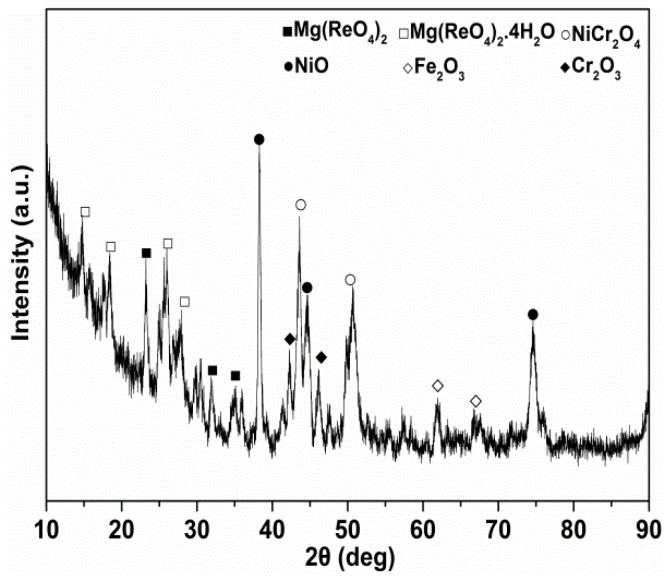
The XRD pattern of worn surface lubricated by PAO containing 0.5 wt% Mg(ReO_4_)_2_ additive after friction test at 600 °C.

**Figure 13:**
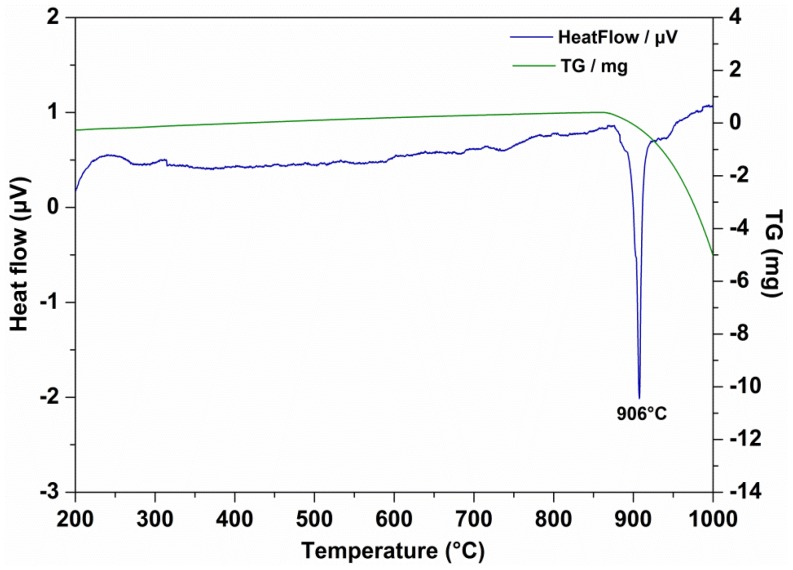
The DTA/TG curves of synthesized Mg(ReO_4_)_2_ powder.

To further determine the chemical stability of Mg(ReO_4_)_2_ as a lubricant, the phase compositions after heat treatment for 10 min at various temperatures were carried out by X-ray diffraction and the patterns are exhibited in Figure 11. Clearly, the phases of Mg(ReO_4_)_2_ and Mg(ReO_4_)_2_·4H_2_O were identifiable after heat treatment, which were basically identical to that at room temperature. It should be noted that no other diffraction peaks exist, indicating that no phase transformation or decomposition occurred at that temperature section. The XRD patterns of worn track surface lubricated by PAO with Mg(ReO_4_)_2_ additive at 600 °C are presented in Figure 12. The XRD diffraction peaks for Mg(ReO_4_)_2_ were observed on the wear track, and the presence of Mg(ReO_4_)_2_ was expected to supply lubrication at high temperature. Very strong NiO and NiCr_2_O_4_ could be detected on the wear track with the Fe_2_O_3_ and Cr_2_O_3_ phases. Except for oxidation and the Mg(ReO_4_)_2_ phase, there were no new peaks, which implied that no reaction took place or too little to be detected by the X-ray diffraction tester. This further proved that the Mg(ReO_4_)_2_ additive was the major contribution essential for the coefficient of friction reduction at elevated temperature, which was in line with the morphology analysis of the worn surface. In general, the hardness of materials was related to its melting point; the higher the melting point, the higher the hardness [30]. Figure 13 shows the DTA/TG curves of Mg(ReO_4_)_2_ compound, and an intensive endothermic peak is located at 906 °C, which was attributed to melting. It was reasonably inferred that Mg(ReO_4_)_2_ with a low melting point would be softened along with the increasing temperature and then form a lubrication layer combined with some natural oxides of the metal on the contact surface. Consequently, the layer would be inclined to be sheared under stress, which eventually results in lowering the friction coefficients.

**Figure 14:**
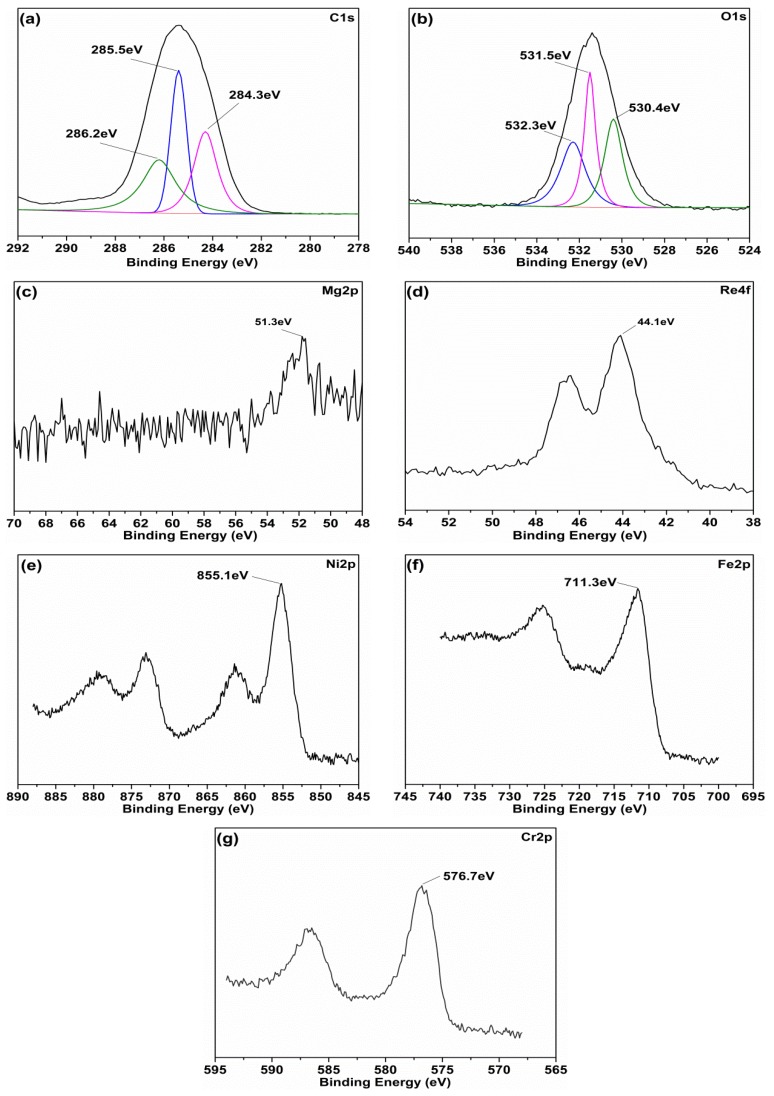
The X-ray photoelectron spectroscopy (XPS) spectras of the typical elements on worn surface lubricated with PAO containing 0.5 wt% Mg(ReO_4_)_2_ after rubbing test at 600 °C: (**a**) C1s; (**b**) O1s; (**c**) Mg2p; (**d**) Re4f; (**e**) Ni2p; (**f**) Fe2p; (**g**) Cr2p.

To better obtain a comprehensive understanding of the protective layer and expose its role in antifriction at high temperature conditions, the XPS analysis of the worn surface of the superalloy disc under oil with Mg(ReO_4_)_2_ additive at 600 °C was investigated as shown in Figure 14. The main binding energy peaks of C1s were located at around 284.3 eV, 285.5 eV, and 286.2 eV, corresponding to C–H bond group, C–C bond group, and C–O bond group in the organic functional groups, respectively [31]. This indicated the decomposed products of PAO and added surfactants were absorbed on the superalloy surface. The O1s was composed of three peaks, and the main peak appeared at 530.4 eV, which could be related to oxygen in the organic matter. An objective peak located at 531.5 eV could be ascribed to natural oxides from the alloy, suggesting that the metallic elements suffered oxidation in the test process. Also, another peak at 532.3 eV associated with oxygen in the ReO_4_^−^ group, indicated that magnesium perrhenate existed on the contact interface [32]. The Mg2p spectrum was detected at 51.3 eV, which could correspond to the chemical state of Mg^2+^. The peak of Re4f appeared at 44.1 eV, which could be assigned to the absorbed Mg(ReO_4_)_2_ additive. The Ni2p peak at 855.1 eV was associated with nickel oxide, and oxidation of iron to Fe_2_O_3_ during the sliding process was confirmed by the binding energy peak at 711.3 eV. Additionally, the Cr2p peak located at 576.7 eV was attributed to the generation of chromium oxide. The existence of native oxide of superalloy showed that the native metallic elements oxidized and developed oxides on the rubbing surface. Nevertheless, these metallic oxides offered little lubrication function at elevated temperature condition, because they are brittle and easily removed under cyclic stress. The XPS analysis revealed that the added Mg(ReO_4_)_2_ additive was deposited first on the metal surface, then was compacted by cyclic stress with native superalloy oxides under the high temperature environment, forming a mixed protective layer on the rubbing interface. The surface protective layer remarkably contributed to the friction-reducing performance of Mg(ReO_4_)_2_ in PAO base oil at elevated temperature.

The shearing property and thermal stability of Mg(ReO_4_)_2_ compound could be interpreted by means of crystal chemistry theory [33]. The double oxide Mg(ReO_4_)_2_ compound could be viewed as two mixed single oxides of MgO and Re_2_O_7_, which have ionic potential of 3.0 and 12.5, respectively. Generally, the cations in oxides are in separated state and fully blocked by surrounding anions on account of large difference in ionic potential, thereby the highly screened cations hardly interact with neighboring cations [34]. Consequently, the double oxides were fairly soft and easy to be sheared at the high temperature range. Additionally, the affinity of ionic species to generate stable compounds improved as the difference in ionic potential increased, and reducing the attractive effect between sliding surfaces led to lower adhesive force across the rubbing interface. Over the temperature range, the Mg(ReO_4_)_2_ compound as lubricating additive, could match oil well without worsening the antifriction and thermal stability of the base oil. Moreover, it could generate a highly compressive layer with natural oxides from the Ni-based alloy on the interface under such an elevated temperature range to sustain a certain amount of applied load. Thus, the magnesium perrhenate could be considered as a promising lubricating oil additive for a broad temperature range application.

## 4. Conclusions

In this paper, the investigation on the tribological performance of PAO base oil mixed with Mg(ReO_4_)_2_ additive with four-ball experimental conditions and ball-on-disc sliding friction tests over a wide temperature range was implemented. In view of the test result analysis, the following conclusions can be drawn:

(1) The Mg(ReO_4_)_2_ compound used as lubricating additive significantly improved the lubricating base oil in the condition of the four-ball test. The coefficient of friction and WSD were reduced by 30.8% and 28.5%, respectively, when the optimum concentration of 0.5 wt% Mg(ReO_4_)_2_ additive was dispersed.
(2) The Mg(ReO_4_)_2_ compound could promote the antifriction property of the base oil at a relatively low temperature and possessed a superb lubrication function at high temperature conditions. The added Mg(ReO_4_)_2_ was deposited on the worn surface and generated a protective layer that improved the contact state of the rubbing interface. This layer formed at the elevated temperature range was mainly made up of Mg(ReO_4_)_2_ compound and some natural oxides from the metal matrix, while the appearance of the complex layer could effectively prevent direct contact between rubbing pairs. The Mg(ReO_4_)_2_ additive had a leading role in friction-reduction as a result of its higher thermal stability and maintaining a shear-susceptible structure with increasing temperature.
(3) The established original characterizations and tribological testing results suggested a tremendous potential for blending proper magnesium perrhenate additive and PAO base oil to obtain a continuous and effective lubrication function over a broad temperature range.

## Figures and Tables

**Table 1:** The physical parameters of poly alpha olefin (PAO6).

Parameter	Density (15 °C, g/cm^3^)	Kinematic Viscosity (mm^2^/s)		Viscosity Index	Pour Point (°C)	Flash Point (°C)
		40 °C	100 °C			
PAO6	0.819	31	5.8	138	−68	246

**Table 2:** The temperature variations of different lubricants under the four-ball tests condition.

Samples	PAO	PAO–SA	0.1 wt%	0.3 wt%	0.5 wt%	0.8 wt%	1.0 wt%
Original temperature (°C)	25.8	26.4	26.7	26.4	26.2	26.1	26.2
Finishing temperature (°C)	63.2	62.9	60.6	57.6	55.8	58.4	59.3
Temperature rise (°C)	37.4	36.5	33.9	31.2	29.6	32.3	33.1
